# Knowledge of risk factors, beliefs and practices of female healthcare professionals towards breast cancer, Morocco

**DOI:** 10.4314/pamj.v10i0.72231

**Published:** 2011-10-13

**Authors:** Samia Ghanem, Meriem Glaoui, Siham Elkhoyaali, Mohamed Mesmoudi, Saber Boutayeb, Hassan Errihani

**Affiliations:** 1 Department of Medical Oncology, National Institute of Oncology of Rabat, Morocco

**Keywords:** Breast cancer, knowledge risk factors, beliefs, breast self examination, mammogram, healthcare professionals

## Abstract

**Background:**

Breast cancer is the most common cancer affecting women in Morocco. Screening for early detection has led to reduction in mortality from the disease. It is known that female healthcare professionals have greater influence on women's positive perception of breast cancer and motivation to practice screening methods for early detection of the disease. This study aims to investigate knowledge of breast cancer risk factors, beliefs about treatment and practice of screening methods among a cohort of female healthcare professionals in Morocco.

**Methods:**

A cross-sectional study was conducted using a self-administered questionnaire to assess the knowledge of breast cancer risk factors, beliefs about treatment and practice of screening methods among 136 female doctors and nurses working in the university hospital of Rabat, Morocco. Stratified random sampling method was employed. Chi square test, analysis of variance and Mantel-Haenszel test were performed in data analysis using SPSS v19.0.

**Results:**

Female doctors were the only professional group that had satisfactory knowledge of risk factors while the nurses had an unsatisfactory knowledge with a mean score of 43%. A half of participants believed that that herbal therapy can cure breast cancer. 75% practice breast self-examination once a month and only 15% have ever had a mammogram. Age, profession and beliefs were not significantly associated with rate of BSE in this study; however this rate is influenced by knowledge of breast cancer risk factors.

**Conclusion:**

Results from this study suggest the need for continuing medical education programs aimed at improving knowledge of breast cancer among the nurses.

## Background

Breast cancer is the most common cancer among women in Morocco. Early detection and treatment of breast cancer is associated with better chance of long-term survival [1], in Morocco, about two-third of patients with this disease present with advanced stages when therapy offers minimal benefit. Female healthcare professionals have greater influence on women′s positive perception of breast cancer and motivation to practice screening methods for early detection of the disease [2]. This is the first study conducted in our country to assess knowledge of breast cancer risk factors, beliefs about treatment and practice of screening methods among female healthcare professionals.

## Methods

This is a cross sectional survey of female healthcare professionals. The aims were to assess level of their knowledge about breast cancer risk factors, beliefs concerning breast cancer treatment and their practice of BSE (breast self examination), CBE (clinical breast examination) and Mammography as screening methods.

### Participants

The study was conducted in May 2010 in the university hospital of Rabat, Morocco. Participants were selected using stratified random sampling method with proportional allocation. Sample size was calculated to achieve 95% confidence and 5% type 1 error. Questionnaires were distributed to participants who returned their questionnaires within 20 minutes. Written informed consent was obtained and assurance of confidentiality of responses was given to each respondent.

### Measurements

Data collection was accomplished using interviewer-administered questionnaires manuscript in Arabic language. The questionnaire was developed by the authors using information on breast cancer from the literature and from questionnaire conducted in the other country. Pre-test was conducted on 15 female healthcare professionals working in the hospital following which some questions were modified to improve clarity. Those that participated in the pre-test were not part of the study.

The questionnaire was in four parts. The first part was to elicit socio-demographic data on age, profession, marital status and qualification of each study participant. Questions relating to knowledge of breast cancer risk factors were asked in the second part. Eight items with different levels of relative risk for breast cancer were included and they were to generate “Yes” or “No” response. Participants’ awareness of breast cancer and early detection methods were also assessed in this section. The third part of the questionnaire assessed participant′s beliefs about breast cancer treatment. In the last part, questions were asked on the practice of BSE, CBE and Mammography among participants. Most of the questions in the third and fourth parts were also designed to elicit similar response of “Yes” or “No”.

### Analysis

Each respondent was scored on knowledge of risk factors based on the number of correct answers (n=8). Out of a total score of 100, percentage score for each participant and mean score for different professional groups were calculated. Knowledge score of the groups of nurses and doctors were compared. A score of >80% was considered as indicating “excellent” knowledge, scores between 60% and 80% was labeled “very good” knowledge. Scores between 40% and 60% represented “good” knowledge while a score of <40% was classified as “poor” knowledge. Response of participants belonging to different professional groups to questions on beliefs and practice of screening methods were also compared.

We used in data analysis the SPSS V19.0. Statistical comparison was carried out using chi-square test for qualitative variables and proportions and analysis of variance for quantitative variables. Stratified analysis was performed on each professional group after which summary chi square and p value were computed. Level of statistical significance was set at P<0.05.

## Results

### Socio-demographic characteristics of study participants

Table 1 shows socio-demographic characteristics of study population. The One hundred and thirty six female healthcare professionals who participated in this study were ninety two nurses (67.6%) and 44 doctors (32.3%), they are all Muslims and Moroccans. Age range of 136 participants was from 22 to 58 years (Mean=34.31). All participants possessed basic professional certificate. Fifty six percent of respondents were married.

**Table 1:** Socio-demographic characteristics of study participants

Variable	Doctors (number)	Nurses (number)	Percentage
**Profession**	44	92	100.0
**Age distribution (n =130)**			
< 20	0	0	0.0
20–29	16	29	34.6
30–39	27	21	36.9
40–49	1	24	19.2
≥ 50	0	12	9.2
**Marital status**			
Married	22	54	55.9
Single	22	38	44.1

### Knowledge of risk factors and awareness of breast cancer

Table 2 and Table 3 show participants responses to questions on breast cancer risk factors and awareness. Twenty-two study participants (16%) had excellent knowledge of risk factors, 33% possessed very good knowledge, 14% had good knowledge while the remaining 38% had poor knowledge of risk factors assessed. 47% among doctors had excellent knowledge and no doctor (0%) was considered as having poor knowledge. All doctors had a satisfactory knowledge and 47% among them had excellent knowledge In contrast, 56.5% among nurses possessed poor knowledge and only 1(1%) had excellent knowledge. Doctors were the only group with satisfactory mean knowledge score of 100%. Mean knowledge score for nurses was 43%.

**Table 2:** Correct knowledge of risk factors among nurses and doctors

Variable	Doctors (n=44) Number (%)	Nurses (n=92) Number (%)	p-value
Increasing age	21(47,7)	44(47,8)	0,983
Positive family history of breast cancer	44(100)	37(40,2)	0,000
First childbirth at the age of ≥ 30 years	21(47,7)	29(31,5)	0,011
Nulliparity	44(100)	19(20,7)	0,000
Early menarche at the age of ≤ 12 years	44(100)	6(6,5)	0,000
Late menopause at the age of ≥ 55 years	44(100)	34(37)	0,000
History of previous benign breast lump	44(100)	60(65,2)	0,000
Current use of oral contraceptive pills	44(100)	63(68,5)	0,000

**Table 3:** Awareness of breast cancer among nurses and doctors

Variable	Doctors (n=44) Number (%)	Nurses (n=92) Number (%)	p-value
Aware of BSE	44(100)	86(93.5)	0.000
Aware of CBE	44(100)	80(87)	0.000
Aware of mammography	44(100)	66(71.7)	0.000
Routine breast cancer screening is necessary for women>40 year	44(100)	88(95.7)	0.004
Breast cancer is a major health problem in our country	44(100)	89(96.7)	0.014
There is sufficient breast cancer awareness in our country	21(47.7)	48(52.2)	0.983

BSE: Breast Self-examination; CBE: clinical breast examination			

Sixty five participants (47%) identified increasing age as a risk factor, 107 (78%) recognized current use of oral contraceptive pills as breast cancer risk factor and 104(76%) recognized history of previous benign breast lump as a risk factor. Other risk factors were recognized by less than three-quarter of participants. Least recognized risk factors was advanced age at first childbirth. Only 35% of participants were able to identify it as risk factor. Difference in knowledge score between doctors and nurses was statistically significant (p<0.001).

One hundred and thirty participants (95%) were aware of BSE. However, a lesser proportion (91%) was familiar with CBE. One hundred and ten participants (80%) were aware of mammography as a screening method for breast cancer. Nearly all participants (97%) agreed that all females above the age of 40 years need to have regular breast cancer screening. Majority of participants believed that breast cancer is a major health problem in our country. The question on the level of breast cancer awareness among the populace elicited varied responses. Half among nurses in this study was satisfied with the level of awareness about breast cancer in the populace whereas only 15.9% among doctors were satisfied with current level of awareness. The difference in the responses of doctors and nurses to this question was statistically significant (P<0.001).

### Beliefs about breast cancer treatment

Responses of participants to questions on beliefs about breast cancer treatment are shown in Table 4. More than 86% of participants believed that breast cancer can be cured if detected early. 65% held the belief that surgery is the most effective method of treatment for breast cancer. Among doctors, majority (81%) believed that herbal or alternative medical therapy cannot cure breast cancer while only 22% of the nurses held similar belief. All doctors and only 40% among nurses believed that breast cancer cannot disappear following prayer without treatment.

**Table 4:** Positive beliefs about breast cancer treatment among nurses and doctors

Variable	Doctors (n=44) Number (%)	Nurses (n=92) Number (%)	p-value
Breast cancer is curable if detected early	44(100)	88(95.7)	0.004
Surgery is the most effective method of treatment for breast cancer	21(47.7)	59(64.1)	0.063
Alternative or herbal medicine cannot cure breast cancer	21(47.7)	20(21.7)	0.000
Alternative or herbal medicine cannot cure breast cancer	44(100)	37(40.2)	0.000

There was no statistically significant difference in response of doctors and nurses to question on whether surgery is the most effective method of treatment for breast cancer or not (p=0.681). Similarly, difference in response of nurses and doctors to question on whether breast cancer can disappear following herbal therapy was not statistically significant (p=0.331) while the responses to the question on whether breast cancer can disappear following prayer without treatment was significant (p<0.001).

### Breast cancer screening practices

Table 5 summarizes breast cancer screening practices among respondents. 75% of participants in this study reportedly practice BSE at least once every month. There was a statistically significant difference in BSE rate between doctors and nurses (p=0.002). Frequency of CBE among participants was rather low with less than one-quarter affirming that they had CBE within the past one year. Majority of those that had CBE did so because of breast symptoms. Only 20 participants (15%) ever had mammogram before this study.

**Table 5:** Practice of screening methods among nurses and doctors

Variable	Doctors (n=44) Number (%)	Nurses (n=92) Number (%)	p-value
Perform BSE at least once a month	44(100)	73(79.3)	0.000
Performed CBE within last 1 year	21(47.7)	24(26.1)	0.001
Had mammography in the past	21(47.7)	15(16.3)	0.000

BSE: Breast self-examination; CBE: clinical breast examination			

Rate of reported BSE practice by participants in this study was not influenced by age (Chi square=7.286, p=0.063), profession (Chi square=2.867, p=0.09) or beliefs about breast cancer treatment (>0.05). However this rate is influenced by knowledge of breast cancer risk factors (Chi square=11.845, p=0.008).

## Discussion

Our study estimated that only 43% of the group nurses in the university hospital of Rabat had good knowledge of breast cancer risk factors. The knowledge of breast cancer risk factors among the nurses is low and is similar to that seen in other developing countries [3,4].

A similar study in Nigeria found an unsatisfactory level of knowledge about breast cancer risk factors among nurses [5]. In Pakistan the level of good knowledge of breast cancer risk factors among nurses working in teaching hospitals of Karachi was low (35%) [6]. in Iran the respondents′ knowledge of risk factors of breast cancer was also not satisfactory [7]. A report from Australia revealed that General Practitioners have limited knowledge of some aspects of breast cancer risk factors with only 25% of those interviewed in the study recognizing increasing age as breast cancer risk factor [4]. In contrast, in United Kingdom, a study of General Practitioners knowledge of breast cancer risk factors showed that more than half of the participants were able to correctly identify risk factors assessed [8]. Nurses in some advanced countries are generally better informed about breast cancer than non-healthcare providers, a report from Jordan, for example, found no significant difference in level of knowledge of risk factors between nurses and schoolteachers [9]. In the United States of America, Powe et al [10] reported that significant number of myths and misperceptions related to breast cancer were prevalent within nursing and non-nursing college students. In addition, few nursing students reported obtaining information on common perception about breast cancer from their course work.

Breast cancer risk factor knowledge among nurses is important so that they can provide appropriate screening recommendations to women with a high risk profile, especially in the context of our country where breast cancer screening is not a national phenomenon. Furthermore, this role has been expanded in some advanced countries with the evolution of specialist breast care nurse [9].

Participants in this study have positive perception of breast cancer treatment. Majority held the belief that early stages of the disease are curable and that surgery is the most effective method of treatment. These findings support are concordant with the report from a similar study among nurses in Nigeria where 89% would agree to have mastectomy should they develop breast cancer [11].

Wrong cultural and religious beliefs about breast cancer are significant factors in late presentation of the disease [12–14]. In this study, majority (81%) of the doctors believed that herbal or alternative medical therapy cannot cure breast cancer and all doctors believed that breast cancer cannot disappear following prayer without treatment.

Among nurses, only 22% believed that herbal or alternative medical therapy cannot cure breast cancer and only 40% among nurses believed that breast cancer cannot disappear following prayer without treatment.

In a study by Mitchell et al [13] strong religious beliefs were found to be common among women in Eastern North Carolina in United States of America. The report showed that a majority believed that God works through doctors to cure breast cancer, the minority who were mainly African-Americans, believed that medical treatment was unnecessary because only God could cure breast cancer.

A significant proportion of the group of nurses in this study believed in efficacy of prayer and traditional or herbal therapy. This calls for concern because such beliefs could have negative effect on their role in creating appropriate awareness about breast cancer in Morocco where majority of those with the disease present with advanced stages [15].

Practice of BSE by participants in this study is insufficient. Only seventy five percent carried out the procedure at least once every month. A lower rate of regular BSE was reported in study conducted in developing nations (Port Harcourt, Iran…), In contrast, higher rates are commonly found among women from developed countries where breast cancer awareness is believed to be better (USA…) [16,17].

The characteristics of the respondents in this study (age, profession, belief about breast cancer treatment) are variable between the two groups: doctors and nurses, There was a no significant association between BSE practice and this characteristics. The principal explanation for low compliance is the lack of education in breast cancer (only 47% of women had a sufficient level of knowledge), in fact, it was found in this study that being knowledgeable of breast cancer is a significant variable in BSE. Similarly, Hyun′s study also reveals that women who are taught to perform BSE have a better level of knowledge about breast cancer [18].

Majority of participants in this study were aware of mammography as a screening method for early breast cancer. However, only 22% among participants ever had mammogram. Reports from developing nations often indicate low rates of mammography screening practice [19,20]. Okobia et al. [19] reported that none of the participants in a study among semi-urban community-dwelling women in Nigeria ever had mammography screening. In contrast, significantly higher rate of mammography screening is often reported among women in advanced countries [17,21].

There are few randomized trials of breast self-examination. One Chinese randomized study and a review of eight other studies failed to show a benefit of breast cancer mortality. Breast self-examination assists women in detecting benign breast lumps and in creating more awareness about breast changes. How to practice BSE is subjective and difficult to be evaluated, two case-control studies suggest that technique is important in BSE and the women who not performing BSE had an increase risk for death or metastatic disease compared to women performing BSE [22].

The adoption of mammography screening has led to reduction in mortality from the disease in women [23,24]. The CBE may modestly improve early detection of breast cancer.

Women with breast cancer in Morocco are relatively younger than the American or the European woman [25]. Therefore, adopting mammography screening guidelines designed for advanced population may not be beneficial in our country, the Performance characteristics of mammography are poor for women younger than 40. In a review of results of 73,335 initial screening mammograms in women aged 35 to 39 years, the younger women have very low breast cancer rates but after mammography experience high recall rates, high rates of additional imaging, and low cancer detection rates [26]. Additionally, compared to older women, there is evidence that younger women have faster growing cancers for which mammographic screening would not be as beneficial [27]. A rapidly growing tumor may be too small to detect at one screening, but already progressed to advanced stage by the subsequent screening.

In the other hand, Morocco has a poor economy and the relative cost of mammography is prohibitive for population screening. Adoption of routine and regular BSE and CBE appear to be more realistic and reasonably methods of breast cancer screening in our country.

## Conclusion

This is the first study to evaluate the level of awareness of breast cancer within the health professionals in Morocco. The results demonstrate that the knowledge of breast cancer risk factors was satisfactory among doctors but inadequate among a large percentage of nurses. There is a need to improve breast cancer content in the nursing teaching programs. It is also important to encourage the nurses to disseminate this knowledge effectively and appropriately within the general population. Significant proportion of participants in this study believed in the role of prayer and alternative medical therapy in the cure of cancer. Programs to educate religious leaders and alternative medical practitioners about breast cancer should be encouraged.
